# Parents’ expectations, preferences, and recall of germline findings in a childhood cancer precision medicine trial

**DOI:** 10.1002/cncr.34917

**Published:** 2023-06-29

**Authors:** Brittany C. McGill, Claire E. Wakefield, Katherine M. Tucker, Rebecca A. Daly, Mark W. Donoghoe, Janine Vetsch, Meera Warby, Noemi A. Fuentes‐Bolanos, Kristine Barlow‐Stewart, Judy Kirk, Eliza Courtney, Tracey A. O’Brien, Glenn M. Marshall, Mark Pinese, Mark J. Cowley, Vanessa Tyrrell, Rebecca J. Deyell, David S. Ziegler, Kate Hetherington

**Affiliations:** ^1^ School of Clinical Medicine University of New South Wales (UNSW) Sydney Sydney New South Wales Australia; ^2^ Kids Cancer Centre Sydney Children’s Hospital Sydney New South Wales Australia; ^3^ Hereditary Cancer Centre Prince of Wales Hospital Sydney New South Wales Australia; ^4^ Prince of Wales Clinical School UNSW Sydney Sydney New South Wales Australia; ^5^ Stats Central UNSW Sydney Sydney New South Wales Australia; ^6^ Children’s Cancer Institute UNSW Sydney Sydney New South Wales Australia; ^7^ Faculty of Medicine and Health Royal North Shore Hospital Sydney New South Wales Australia; ^8^ Familial Cancer Service Westmead Hospital Sydney New South Wales Australia; ^9^ Sydney Medical School Westmead Institute for Medical Research Sydney New South Wales Australia; ^10^ Division of Paediatric Hematology/Oncology/Bone Marrow Transplantation British Columbia Children’s Hospital and Research Institute Vancouver British Columbia Canada

**Keywords:** genomic medicine, germline mutation, hereditary cancer syndromes, neoplasms, pediatrics, precision medicine

## Abstract

**Background:**

Germline genome sequencing in childhood cancer precision medicine trials may reveal pathogenic or likely pathogenic variants in cancer predisposition genes in more than 10% of children. These findings can have implications for diagnosis, treatment, and the child’s and family’s future cancer risk. Understanding parents’ perspectives of germline genome sequencing is critical to successful clinical implementation.

**Methods:**

A total of 182 parents of 144 children (<18 years of age) with poor‐prognosis cancers enrolled in the Precision Medicine for Children with Cancer trial completed a questionnaire at enrollment and after the return of their child’s results, including clinically relevant germline findings (received by 13% of parents). Parents’ expectations of germline genome sequencing, return of results preferences, and recall of results received were assessed. Forty‐five parents (of 43 children) were interviewed in depth.

**Results:**

At trial enrollment, most parents (63%) believed it was at least “somewhat likely” that their child would receive a clinically relevant germline finding. Almost all expressed a preference to receive a broad range of germline genomic findings, including variants of uncertain significance (88%). Some (29%) inaccurately recalled receiving a clinically relevant germline finding. Qualitatively, parents expressed confusion and uncertainty after the return of their child’s genome sequencing results by their child’s clinician.

**Conclusions:**

Many parents of children with poor‐prognosis childhood cancer enrolled in a precision medicine trial expect their child may have an underlying cancer predisposition syndrome. They wish to receive a wide scope of information from germline genome sequencing but may feel confused by the reporting of trial results.

## Abstract 2

Parents of children with poor‐prognosis cancer enrolled in a precision medicine trial may overestimate the likelihood of their child receiving a clinically relevant germline finding and may feel confused by the reporting of trial results. We discuss the implications of these data for informed‐consent and return of results practices in precision medicine trials.

## INTRODUCTION

Precision medicine trials are forging ahead worldwide, with the aim of improving treatment options for children with cancer.^1^, ^2^, ^3^, ^4^, ^5^, ^6^ Oncology precision medicine can be defined as the use of next‐generation sequencing (NGS) to conduct comprehensive analysis of (1) tumor tissue, to inform diagnosis, predict prognosis, and identify actionable somatic variants, and (2) germline tissue, to identify pathogenic or likely pathogenic (P/LP) variants in genes that confer heritable predisposition to cancer.^7^

Initial studies indicated that approximately 1 in 10 children with cancer harbor P/LP variants in cancer predisposition genes.^8^, ^9^ Recent studies offering germline genome sequencing as part of precision medicine initiatives indicate that the rate of cancer‐predisposing variants may be as high as 18%.^1^, ^10^, ^11^ These findings may change the child’s cancer management, inform understanding of future cancer susceptibility in the child and genetic relatives, and guide long‐term cancer surveillance protocols and family‐planning decisions.^12^, ^13^ The added value of germline genome sequencing in this context includes the identification of germline variants in children and families who do not meet clinical testing guidelines,^14^ the potential for paired tumor–germline testing to inform understanding of tumor biology and to enhance treatment choices,^15^ and the increased cost‐effectiveness and speed of return of results as NGS advances.^15^

Studies indicate that parents of children with cancer are interested in genome sequencing, even at the stressful times of cancer diagnosis and relapse, and report being motivated by a desire to understand the causes of their child’s cancer^16^, ^17^, ^18^, ^19^ and hope that research participation will be helpful for future patients if not for their own child.^17^, ^19^, ^20^, ^21^ However, parents may have high expectations of genome sequencing, which may not align with the likelihood of their child receiving clinically relevant results.^21^, ^22^, ^23^, ^24^ Parents may overestimate the heritable risk of their child’s cancer on the basis of their incomplete (or inaccurate) knowledge of their family’s history of cancer.^25^, ^26^ Family history may not actually be a strong predictor of underlying cancer predisposition syndromes (CPSs) in children because of variability in penetrance, high rates of de novo variants, germline mosaicism, and the absence of family history of cancer in young families.^8^, ^14^, ^27^

Parents’ preferences for the scope of information returned to them from germline genome sequencing is important to understand in the highly stressful context of poor‐prognosis pediatric cancer. Current evidence indicates that parents of children with cancer, and other rare disorders, want to learn about results that indicate a risk for unrelated clinically actionable conditions (incidental findings) as well as findings of uncertain significance.^21^, ^28^, ^29^, ^30^ However, returning variants of uncertain significance (VUSs) from genome sequencing may generate uncertainty and disappointment.^31^, ^32^ Parents may also have a limited understanding of genomic concepts,^20^ including the distinction between somatic and germline testing,^33^ and oncologists may feel ill prepared to return results of genomic testing.^34^ These challenges may be compounded by emotional distress and the lack of embedded genetic counseling and psychosocial support in many trials. Building on growing evidence informing best‐practice informed consent for pediatric precision medicine,^20^, ^33^, ^35^, ^36^ we aimed to answer the following:

1. When they enroll their child with a poor‐prognosis cancer in a precision medicine trial, what are parents’ expectations regarding the likelihood of germline genome sequencing identifying clinically relevant results, and what factors influence their expectations?
2. What are parents’ preferences for the scope of information returned to them from germline genome sequencing?
3. What do parents recall about germline findings after receipt of their child’s trial results?

## MATERIALS AND METHODS

### Study context

The Precision Medicine for Children with Cancer (PRISM) study is a multicenter precision medicine clinical trial for children and young persons (≤21 years of age) with poor‐prognosis malignancies (expected likelihood of survival < 30%) at diagnosis or relapse, embedded in Australia’s Zero Childhood Cancer program (Australian New Zealand Clinical Trials Registry: NCT03336931).^1^, ^37^ PRISM aims to assess the feasibility and clinical utility of a molecular profiling platform to identify clinically significant somatic and germline molecular features relevant to the child’s cancer diagnosis, treatment, or prognosis (Figure S1).^1^ Families are consented to PRISM by their child’s treating clinician and can choose to opt out of being informed of clinically relevant germline findings.

For the germline samples, a large panel of 161 cancer predisposition genes with an established impact on cancer risk was analyzed.^1^ P/LP germline variants were considered clinically relevant if they were determined to be (1) informative for the family (in terms of the tumorigenesis of the malignancy or reproductive decision‐making) and/or (2) clinically actionable for the child (in terms of risk of toxicities and second malignancies) and/or the family (with regard to cascade testing and initiation of cancer risk reduction/surveillance). The analysis pipeline was designed to avoid detecting variants unrelated to cancer (incidental findings).

After review by the PRISM Molecular Tumor Board (MTB), which included oncologists, pathologists, geneticists, genetic counselors, basic scientists, bioinformaticians, and study managers, a report was generated for the child’s treating clinician summarizing the presence or absence of clinically relevant somatic and germline findings. Any germline VUSs identified were flagged by the MTB cancer genetics working group for periodic review to reassess reportability but were not shared with the child’s clinician. Results were delivered to parents by the child’s treating clinician in a face‐to‐face consultation as part of clinical care. Referral to local cancer genetics services was recommended in the PRISM report for any family whose child received a germline finding; referral was then provided at the treating clinician’s discretion.

### Participants

We invited all parents of children enrolled in PRISM to participate in a mixed‐methods psychosocial study (PRISM‐Impact; Figure S1). Parents completed questionnaires at PRISM enrollment (time 0; T0) and after the return of PRISM results (T1), and were also invited to an optional qualitative interview at T1. PRISM and PRISM‐Impact received institutional board approval (17/02/15/4.06; HREC/17/HNE/29) and were conducted in accordance with the Declaration of Helsinki.

### Design

We used a concurrent quantitative‐dominant mixed‐methods approach. This involved collecting quantitative and qualitative data at the same time, with the qualitative interview designed to enhance understanding of the questionnaire data at the analysis phase.^38^

### Procedure

A trained psychosocial researcher conducted an intake call with parents 2 weeks after they opted in to PRISM‐Impact to assess their questionnaire preferences (online/hard copy). The baseline (T0) questionnaire was sent immediately after the intake call. We sent the second questionnaire (T1) once clinical records indicated the clinician had delivered the child’s PRISM results to their parents. For the optional qualitative interview, interest was assessed during the intake call. Those who opted in were contacted at T1 to arrange and complete the interview over the phone. Interviews were audio recorded and transcribed verbatim.

### Data collection

#### Questionnaires

##### Participant characteristics (T0)

We assessed parents’ age, gender, marital status, cultural background, first language, religion, education level, and employment status. We also accessed the child’s clinical records to confirm the child’s diagnosis, family history of cancer (recorded by the clinician as “yes”: at least one first/second‐degree relative with cancer; “no”; or “not assessed”^1^), and the child’s PRISM results.

##### Expectations regarding germline findings (T0; purposively developed item)

We asked parents to rate their perception of the likelihood that the “PRISM tests will show a change in your child’s genes that runs in families, or can be passed from generation to generation (a ‘mutation’)” on a five‐point Likert scale ranging from 1 (very likely) to 5 (very unlikely).

##### Past genetic/genomic testing in family members (T0; purposively developed item)

We asked parents to indicate whether anyone in their immediate family had ever had genetic or genomic testing outside of the PRISM trial (yes/no/unsure).

##### Perceived genetics knowledge (T0; purposively developed item)

We asked parents to rate their knowledge of genetics, compared to the average person, on a Likert scale, with 1 = below average, 2 = about average, and 3 = better than average.

##### Preferences regarding germline genome sequencing results (T1)

We asked parents to indicate their preferences to receive information that their child or family might be at increased risk of (1) “cancer that is treatable/preventable,” (2) “cancer that is not treatable/preventable,” (3) “other diseases that are treatable/preventable,” (4) “other diseases that are not treatable/preventable,” and (5) “information about your child’s/family’s health, but doctors do not know if it would cause any diseases” (i.e., variants of uncertain significance). Response options were “would have wanted” and “would not have wanted.”^21^

##### Recall of germline findings (T1; purposively developed item)

We asked parents to indicate their response (yes/no/unsure) to the question “Did the genetic testing done as part of PRISM find any changes in your child’s genes, which may run in families or be inherited across generations, that were reported back to you and your child’s doctors?”

#### Semistructured qualitative interview

Trained psychosocial researchers with no prior clinical relationship with the participants conducted the interviews. We explored parents’ understanding of whether their child received a clinically relevant germline finding from PRISM. Any parents who reported that their child received a germline finding were asked about this experience (Table S1).

### Data analysis

#### Questionnaires

We used the Statistical Package for the Social Sciences 26.0 to perform quantitative analyses. We used descriptive statistics to summarize participant characteristics. We fit an ordinal logistic regression model to assess the association between participants’ characteristics (family cancer history, perceived genetics knowledge, and family history of genetic/genomic testing) and their expectations of the likelihood of receiving germline findings.^2^

#### Semistructured qualitative interview

The interviews were coded by two female researchers (B.C.M. and R.A.D.) with postgraduate training in psychology and research methods. Neither coder had a preexisting clinical relationship with the parents interviewed. By using thematic analysis,^39^ B.C.M. reviewed the transcripts and then developed an initial coding system guided by the study aims. B.C.M. and R.A.D. independently coded a randomly selected subset of four interviews line by line, with discrepancies resolved via discussion until high intercoder agreement (>80%) was reached. B.C.M. then coded all interviews and developed the themes and subthemes, which were critically appraised via ongoing discussion with R.A.D. Then, B.C.M. extracted illustrative quotes, which were reviewed by the PRISM‐Impact team.

## RESULTS

### Participants

Tables 1 and 2 summarize participants’ characteristics. All 182 parents participating in PRISM‐Impact had consented to receiving clinically relevant germline findings. Our records indicated that 74% (135 of 182) of these parents had been notified of their child’s PRISM results and any recommendations by their clinician, which made them eligible to receive our T1 questionnaire. For 17 of these parents (17 of 135; 13%) of 12 children, the PRISM study had revealed a clinically relevant germline finding for their child.

**TABLE 1:** Characteristics of parents participating in PRISM‐Impact.

Characteristic	All participating parents (*N* = 182)	Interviewed participants (*N* = 45)
Age, years		
Mean (SD)	41.6 (7.4)	43.1 (7.7)
Range	23–67	29–67
Gender, No. (%)		
Female	114 (62.6)	32 (71.1)
Male	68 (37.4)	13 (28.9)
Marital status, No. (%)		
Never married/de facto	3 (1.6)	0
Currently married/de facto	158 (86.8)	38 (84.4)
Separated/divorced/previous de facto	20 (11.0)	7 (15.6)
Widowed	1 (0.5)	0
Highest level of education, No. (%)		
High school	32 (17.6)	5 (11.1)
Apprenticeship	8 (4.4)	1 (2.2)
Certificate/diploma	52 (28.6)	15 (33.3)
University undergraduate	53 (29.1)	16 (35.6)
University postgraduate	37 (20.3)	8 (17.8)
Employment status, No. (%)		
Employed full‐time	82 (45.1)	19 (42.2)
Employed part‐time	39 (21.4)	10 (22.2)
Employed casual	9 (4.9)	2 (4.4)
Not employed	19 (10.3)	7 (15.6)
Home duties	32 (17.6)	7 (15.6)
Missing	1 (0.5)	0
Cultural background, No. (%)		
Western/European	140 (76.9)	36 (80.0)
Other	37 (20.3)	8 (17.8)
Missing	5 (2.7)	1 (2.2)
Aboriginal or Torres Strait Islander, No. (%)		
Yes	1 (0.5)	1 (2.2)
No	180 (98.9)	44 (97.8)
Missing	1 (0.5)	0
Religion, No. (%)		
No religion	81 (44.5)	20 (44.4)
Christianity	86 (47.3)	20 (44.4)
Buddhism	2 (1.1)	0 (0.0)
Islam	5 (2.7)	2 (4.4)
Hinduism	4 (2.2)	0
Judaism	1 (0.5)	0
Other religion	2 (1.1)	2 (4.4)
Missing	1 (0.5)	1 (2.2)
Perceived genetics knowledge^a^		
Below average	48 (26.4)	6 (13.3)
About average	104 (57.1)	32 (71.1)
Better than average	28 (15.4)	7 (15.6)
Missing	2 (1.1)	0
Past genetic/genomic testing in immediate family^b^		
Yes	17 (9.3)	4 (8.9)
No	149 (81.9)	38 (84.4)
Unsure	15 (8.2)	3 (6.7)
Missing	1 (0.5)	0
Family history of cancer, No. (%)^c^		
Yes	26 (14.3)	7 (15.6)
No	59 (32.4)	17 (37.8)
Not assessed/recorded	97 (53.3)	21 (46.7)
Time from consent and T0, weeks		
Mean (SD)	7.2 (4.6)	
Range	1.3–29.7	
Time from return of results and T1, weeks		
Mean (SD)	14.0 (9.8)	
Range	0.7–45.8	

Abbreviations: PRISM, Precision Medicine for Children with Cancer; PRISM‐Impact, the psychosocial substudy running alongside the PRISM study; T, time.

^a^ Self‐reported at T0.

^b^ Other than the PRISM study; self‐reported at T0.

^c^ Information obtained during a clinical consultation. “Not assessed/recorded” includes missing data and cases where the clinician recorded that they did not assess the family history of cancer.

**TABLE 2:** Characteristics of children whose parents participated in PRISM‐Impact.

Characteristic	Children of all participating parents (*N* = 144)	Children of interviewed participants (*N* = 43)
Age at diagnosis, years^a^		
Mean (SD)	8.0 (5.5)	7.9 (5.6)
Range	0–17	0–17
Age at PRISM enrollment, years		
Mean (SD)	9.1 (5.5)	9.0 (5.7)
Range	0–18	1–17
Initial diagnosis, No. (%)		
Sarcoma	41 (28.5)	13 (30.2)
CNS	57 (39.6)	16 (37.2)
Leukemia/lymphoma	21 (14.6)	4 (9.3)
Neuroblastoma	14 (9.7)	4 (9.3)
Other	11 (7.6)	6 (14.0)
Number of relapses, No. (%)		
0	63 (43.8)	19 (44.2)
1	65 (45.1)	19 (44.2)
>1	16 (18.7)	5 (11.6)
PRISM report recommendations, No. (%)^b^		
No recommendations	37 (27.4)	11 (25.6)
Change in therapy	78 (57.8)	27 (62.8)
Clinically relevant germline finding	6 (4.4)	1 (2.3)
Change in therapy and change in diagnosis	3 (2.2)	0
Change in therapy and clinically relevant germline finding	11 (7.6)	4 (9.3)
Clinically relevant germline findings, No. (%)^b^		
Yes	17 (12.6)	5 (11.6)
No	118 (87.4)	38 (88.4)

Abbreviations: CNS, central nervous system; PRISM, Precision Medicine for Children with Cancer; PRISM‐Impact, the psychosocial substudy running alongside the PRISM study.

^a^ The age restriction is based on the child’s age at the date of consenting to PRISM, whereas the age summarized in the table is that reported by the parent at baseline. Hence, it is possible to include children aged 18 years at baseline if they had their birthday between PRISM consent and baseline.

^b^ As listed on the PRISM study report. Only includes reports that had been shared with parents (*n* = 135) at the time of data analysis.

### Research question 1: Parents’ expectations regarding germline findings and factors influencing their expectations

Of the 182 parents who completed the T0 questionnaire, 176 completed the item about their expectations. At PRISM enrollment, most parents (110 of 176; 63%) reported that they believed it was at least “somewhat likely” that their child would receive a clinically relevant germline finding. Thirty‐eight percent of parents (66 of 176) reported that it was “unlikely” or “very unlikely.”

**FIGURE 1:**
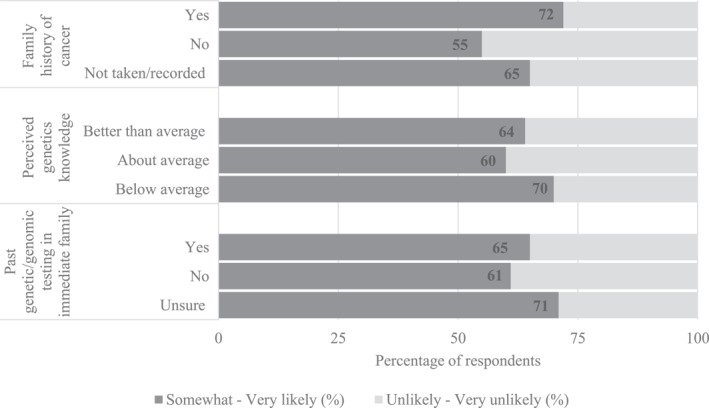
Percentage of respondents by characteristic who reported that it was at least “somewhat likely” that their child would receive a clinically relevant germline finding from PRISM.

Of the parents with family cancer histories, 72% considered it at least somewhat likely that their child would receive a germline finding from PRISM, compared to parents without family cancer histories (55%) and those whose family cancer histories were not assessed/recorded (65%; Figure 1). However, in multivariable ordinal regression, the adjusted comparison between groups was not significant (odds ratio [OR], 0.49, *p* = .119, 95% CI, 0.20–1.20; OR, 0.64, *p* = .298, 95% CI, 0.28–1.48). Similarly, we did not observe significant differences in parents’ expectations regarding the likelihood of their child receiving a germline finding according to their perceived knowledge of genetics (OR, 0.85, *p* = .681, 95% CI, 0.39–1.86; OR, 1.29, *p* = .577, 95% CI, 0.53–3.10) nor whether they reported other genetic/genomic testing in their family (OR, 0.62, *p* = .333, 95% CI, 0.23–1.63; OR, 0.88, *p* = .846, 95% CI, 0.23–3.34).

At interview (Table 3), parents’ expectations tended to be informed by a belief that cancers in their family may be heritable. One parent described how their expectations led to confusion when no clinically relevant findings were returned:

I’ve been told that it’s not a hereditary thing. This is where I get confused, because I know on my husband’s side there have been cancers… (mother of a 12 year old, central nervous system [CNS] diagnosis; no clinically relevant germline finding).

**TABLE 3:** Parents’ experiences of germline genome testing, with illustrative quotes.

Theme	Subtheme	Illustrative quote(s)
Expectations	Beliefs about cancer running in the family	I actually found out [child]’s full cousin had a brain tumour as well, so in the back of my mind I’m thinking “is this genetic?”… So, when they mentioned they were going to do a genetic test, I was really keen on getting that back (mother of a 14 year old, CNS diagnosis; no clinically relevant germline finding).
		[My dad’s] brother died from a brain tumour. It was a familial brain tumour, or they think it was. It was nearly 20 years ago, at the introduction of familial genetic testing. My aunty sent me through that paperwork when she found out that [child] had cancer (mother of a 16 year old, CNS diagnosis; clinically relevant germline finding).
Recall of germline findings	Confident recall	The germline mutation is in [gene]. And at the moment there’s nothing they can do about [gene] and it’s so variable that it just depends how it manifests essentially, going forward (mother of a 3 year old, leukemia/lymphoma diagnosis; clinically relevant germline finding).
		They sent us a letter to say that they didn’t find like a hereditary connection. So yeah, that was good as well. That was useful information to have. So, they said that they don’t see a potential issue in future children (father of a 1 year old, sarcoma diagnosis; no clinically relevant germline finding).
		We were told that, no, it wasn’t hereditary. It wasn’t genetic. It was just bad luck she got it (father of a 4 year old, CNS diagnosis; no clinically relevant germline finding).
	Uncertainty or confusion	I don’t know if I got the information though…actually, I did get some results. I did get some results which are probably in with all my other notes. Cause’ [the PRISM team] did ask if I wanted to know the results or not. I can’t remember if I actually said I wanted to or not (mother of a 16 year old, leukemia/lymphoma diagnosis; no clinically relevant germline finding).
		I know there is something wrong with his genes. There was a mutation, and there’s no medicine at the moment, and maybe there will be. I don’t know if there is any more I can get with my level of understanding (mother of an 11 year old, leukemia/lymphoma diagnosis; no clinically relevant germline finding).
		It was only a real quick conversation with the doctor while we were talking about something else, like, “oh we didn’t find anything there.” …the genetic testing for any predisposed cancer—did they do any of that? I just wanted to be told something (mother of a 5 year old, “other” diagnosis; no clinically relevant germline finding).
	Awaiting consultation with cancer genetics services	The genetic change that they did find is not related to [child]’s tumour and apparently it’s not associated with [child’s] cancer. But it’s associated with another type of cancer.... So, to be honest, they don’t know how to interpret it…it’s something that we are going to discuss with the genetic counsellor… (mother of a 2 year old, neuroblastoma diagnosis; clinically relevant germline finding).
		At the moment it’s about getting through treatment. Yes, it’s another issue that [child] may have to cross later on. For right now, we need to deal with now (mother of a 16 year old, CNS diagnosis; clinically relevant germline finding).
Impact of germline genome testing	Positive impact: Valued information	It scared me, but I’d rather know…the more information I know about myself and their father, the better I can deal with it…then I can know more, in terms of my kids. If you know [the cancer predisposition syndrome] is there, you can make choices based on it (mother of a 16 year old, CNS diagnosis; clinically relevant germline finding).
	Negative impact: Personal emotional consequences	It’s a mix between kind of relief, that we know what it is, and then horror and guilt I guess. You know, I can’t believe that so much could go wrong in such a tiny person…trying to work out if it’s something you’ve done which obviously it’s not but.... Yeah, it’s just a mixed bag of feelings (mother of a 3 year old, leukemia/lymphoma diagnosis; clinically relevant germline finding).
	Negative impact: Emotional consequences for the family	That was a little stressful on not only us, but the rest of my family. My brothers and sisters were worried because they’ve got kids around the same age.... (father of a 1 year old, “other” diagnosis; clinically relevant germline finding).

*Note*: We conducted some minor edits of quotes to improve readability; for example, removal of filler words (e.g., “um”) and repeated words/phrases (e.g., “you know, you know”).

Abbreviation: CNS, central nervous system.

### Research question 2: Parents’ preferences regarding germline genome sequencing results

**FIGURE 2:**
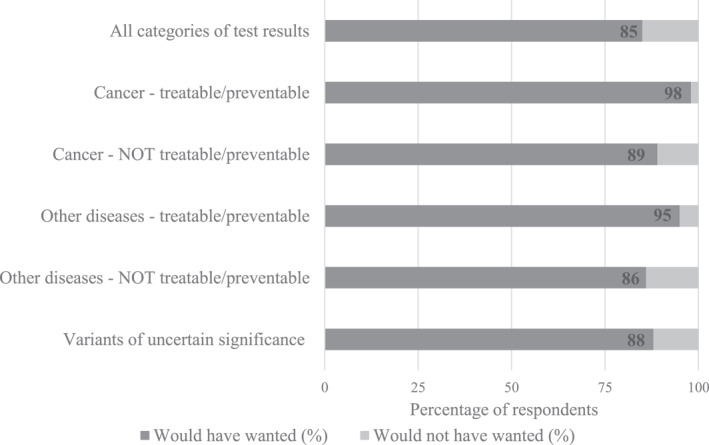
Percentage of respondents reporting their preference (“would have wanted” or “would not have wanted”) for each possible category of test result from genome sequencing. *n* = 107 parents completed these items at time 1.

Most parents indicated that they “would have wanted” to receive all categories of test results from germline genome sequencing (91 of 107; 85%), including variants of uncertain significance (94 of 107; 88%; Figure 2).

Interviewees whose child received a clinically relevant germline finding reflected on the value of this information for both their child and wider family. As one parent explained:

It’s beneficial to know which genes are the ones that have caused the havoc…it’s opened the door to teach the whole family (mother of a 3 year old, leukemia/lymphoma diagnosis; clinically relevant germline finding).

### Research question 3: Parents’ recall of germline findings

Of the 135 parents who had been notified about their child’s PRISM results, 98 completed the T1 questionnaire, including the item assessing their recall of whether their child had received a clinically relevant germline finding. Of these parents, 33 of 98 (33%) recalled receiving a germline finding from the PRISM study. Twenty‐eight (28 of 98; 29%) did not recall receiving a germline finding, and 37 of 98 (38%) reported they were unsure.

**FIGURE 3:**
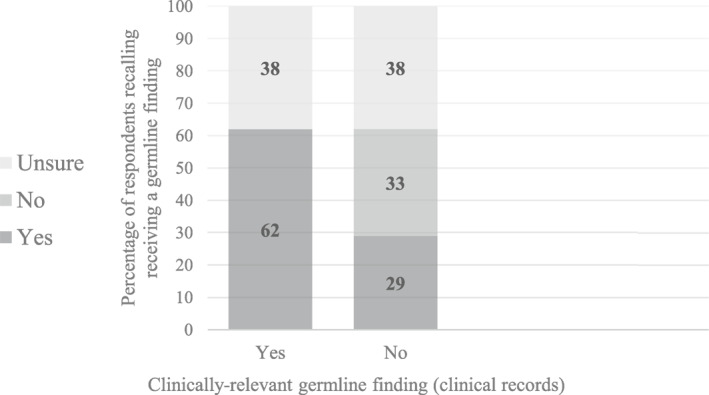
Concordance between parents’ self‐reported recall of whether their child received germline findings (unsure/no/yes) and clinical records of whether a clinically relevant germline finding was delivered to parents (yes/no).

In terms of the concordance between parents’ recall and clinical records, of the 13 parents whose child received a germline finding and answered the recall item, 8 of 13 (62%) accurately recalled that they had received a germline finding. Five (5 of 13; 38%) indicated that they were unsure. Of the 85 parents whose child did not receive a germline finding and answered the recall item, 28 of 85 (33%) accurately recalled that they did not receive a germline finding. Twenty‐five (25 of 85; 29%) inaccurately recalled that they had received a germline finding, whereas 32 of 85 (38%) were unsure (Figure 3).

At interview, some parents confidently and accurately recalled whether their child had received a clinically relevant germline finding. However, some interviewees expressed confusion or uncertainty regarding receipt of germline findings:

They did say “gene mutations” but I wasn’t sure whether it was predisposition or it was mutation in an actual tumour of the cancer itself… (father of an 8 year old, neuroblastoma; no clinically relevant germline finding).

Parents whose child had received a germline finding described both positive and negative impacts of germline genome sequencing. The findings were generally valued by parents, a few of whom had already proceeded with their own genetic testing:

Yes, it’s scary…but I’d much rather know than not know (mother of a 16 year old, CNS diagnosis; clinically relevant germline finding).

## DISCUSSION

Our prospective mixed‐methods study allowed both breadth and depth of understanding of parents’ perspectives on germline genome sequencing for their child with poor‐prognosis cancer. Most parents in our study believed it was at least somewhat likely that their child would receive a clinically relevant germline finding from the PRISM trial. Given that 10%–18% of children with cancer have an underlying genetic predisposition,^1^, ^10^, ^11^ our study suggests that parents overestimate this likelihood. Accordingly, one of the focuses of informed‐consent conversations in this setting should be managing families’ expectations, possibly supported by early involvement of cancer genetics services. Based on our qualitative evidence that parents’ expectations may be influenced by their lived experience or a cancer family history, exploration of parents’ beliefs about underlying cancer predisposition in their family may provide an important opportunity for proactive education and reduction in unnecessary anticipatory distress for many families.^40^

Most parents in our study wanted to receive a broad range of results from germline genome sequencing, including incidental findings and VUSs. This aligns with previous literature that shows that parents want the full scope of their child’s genomic health information, even if the clinical implications are uncertain.^21^, ^28^, ^41^ Our findings should be interpreted with the caveat that our questionnaire provided a general definition of VUSs and no education about their implications. Nevertheless, the tension between parents’ information preferences and current guidelines, which do not recommend the return of VUSs in research settings, requires closer examination.^32^

Parents’ recall of their child’s germline findings from PRISM was variable. Over half of parents whose child received a clinically relevant germline finding demonstrated accurate recall, yet some were unsure. The potential consequences of misunderstanding genomic test results delivered in the context of research trials may include failure to take up referrals to cancer genetics services, which may delay cascade testing of other family members and initiation of appropriate surveillance procedures.^35^ Our data speak to the important role of genetics education and repeated genetic counseling consultations, including at the time of consent and in the return of germline findings.^33^, ^35^ Education and training for clinicians, some of whom have previously described a lack of skills and confidence in communicating genomic results to families,^24^, ^34^, ^42^ are also increasingly important with the continued uptake of genome sequencing in childhood cancer care.

Many parents of children who did not receive a clinically relevant germline finding inaccurately self‐reported that they had received a germline finding or expressed uncertainty about the findings received. Our qualitative data suggest that this may reflect parents’ general confusion about the reporting of the trial results, including the distinction between somatic and germline results. In PRISM, results were disclosed verbally by the child’s treating clinician, which may be suboptimal for comprehension and retention. Our findings support potential changes to informed‐consent and delivery of results practices within the Zero Childhood Cancer program, including provision of hard‐copy reports and the integration of genetic counseling services in the return of germline findings to families. Interventions may include separate, two‐stage information and consent processes for somatic and germline genome testing and the development of more detailed informed‐consent procedures for germline genome testing supported by educational video resources.

Our results should be interpreted with the caveat that there was often a delay between parents’ receipt of their child’s PRISM findings and completion of our follow‐up questionnaire, which may have affected their recall of the results received. We also likely assessed parents’ perspectives before some families were linked in with cancer genetics services. Clinicians may have delayed referral for some families if the CPS diagnosis did not have immediate implications for the child’s acute cancer treatment. Follow‐up data are required to assess the patterns of referral to cancer genetics services and parents’ uptake of these consultations and subsequent cascade testing. Another caveat is that all children in the PRISM trial had a poor prognosis. This may limit the generalizability of our findings to patient groups with better prognoses, whose parents may have different expectations of and experiences with testing.

In addition, our sample included an overrepresentation of mothers and highly educated English‐speaking parents. Our sample also underrepresented parents with self‐perceived “below average” knowledge of genetics, who may benefit most from interventions to improve understanding of genome sequencing. However, we acknowledge that the assessment of genetics knowledge via a simple self‐assessed item has limitations in terms of evaluating genetics literacy, given that perceived and objective understanding do not always correlate. The use of validated scales, such as the Precision in Pediatric Sequencing Knowledge Questionnaire,^43^ may be useful in future studies. Because all parents in our study consented to receive germline findings, we were unable to capture the perspectives of declining families, as has been explored elsewhere.^44^ Also, the family history data accessed via clinical records were incomplete. Revisiting a detailed family history, facilitated by clinical prediction tools, will help to identify children requiring referral to clinical genetics.^13^, ^45^ Finally, although we took reasonable steps to ensure the trustworthiness of our qualitative analysis, we acknowledge that researcher bias is an inherent limitation of this methodology.

Parents of children with cancer enrolled in a precision medicine trial desire a wide scope of results to be returned to them from germline genome sequencing. They may have inflated expectations of their child having an underlying predisposition to cancer and may experience confusion in the early period following receipt of trial results. The findings highlight the need for informed‐consent conversations focused on managing parents’ expectations and enhancing their understanding of the benefits and limitations of germline genome sequencing, in conjunction with genetics education and counselor‐supported return of results, to improve families’ experiences of pediatric precision medicine.

## AUTHOR CONTRIBUTIONS

**Brittany C. McGill**: Conceptualization, formal analysis, writing–original draft, and writing–review and editing. **Claire E. Wakefield**: Conceptualization, funding acquisition, methodology, and writing–review and editing. **Katherine M. Tucker**: Conceptualization and writing–review and editing. **Rebecca A. Daly**: Conceptualization, data curation, and writing–review and editing. **Mark W. Donoghoe**: Conceptualization, formal analysis, and writing–review and editing. **Janine Vetsch**: Conceptualization and writing–review and editing. **Meera Warby**: Conceptualization and writing–review and editing. **Noemi A. Fuentes‐Bolanos**: Conceptualization and writing–review and editing. **Kristine Barlow‐Stewart**: Conceptualization and writing–review and editing. **Judy Kirk**: Conceptualization and writing–review and editing. **Eliza Courtney**: Conceptualization and writing–review and editing. **Tracey A. O’Brien**: Conceptualization and writing–review and editing. **Glenn M. Marshall**: Conceptualization and writing–review and editing. **Mark Pinese**: Conceptualization and writing–review and editing. **Mark J. Cowley**: Conceptualization and writing–review and editing. **Vanessa Tyrrell**: Conceptualization and writing–review and editing. **Rebecca J. Deyell**: Conceptualization and writing–review and editing. **David S. Ziegler**: Conceptualization and writing–review and editing. **Kate Hetherington**: Conceptualization, project administration, and writing–review and editing.

## CONFLICT OF INTEREST STATEMENT

David S. Ziegler has been a consultant for Bayer, Day One, FivepHusion, Accendatech, Amgen, Novartis, AstraZeneca Australia, Norgine, and Alexion Pharmaceuticals. The other authors declare no conflicts of interest.

## Supporting information

Supplementary Material S1

Figure S1

Table S1

## Footnotes

1 Instances where the clinician indicated that family history was “not assessed” were combined with missing data and recoded to the category “not assessed/recorded.”

2 The PRISM study is ongoing; for this study, we analyzed data from parents of children aged <18 years enrolled in PRISM between September 2017 and July 2021, who returned PRISM‐Impact T0 questionnaires up until August 11, 2021, excluding those who were still participating in the study but had not yet reached T1.
