# Supplementary material for: Parents’ expectations, preferences, and recall of germline findings in a childhood cancer precision medicine trial
Source: Cancer. 2023 Jun 29;129(22):3620–32. doi: 10.1002/cncr.34917 (PMC10952780; doi:10.1002/cncr.34917)
Supplement: Supplementary file 1 — Supplementary Material S1 [file CNCR-129-3620-s002.docx]

**Supplementary Table 2.** Factors associated with parents’ decision to participate in PRISM-Impact

| **Variable** |  | **OR (95% CI)** | ***p*-value** |
| --- | --- | --- | --- |
| Child’s cancer type (vs CNS) | Sarcoma | 0.76 (0.43, 1.35) | 0.049 |
|  | Leukaemia / Lymphoma | 0.40 (0.20, 0.80) |  |
|  | Neuroblastoma | 0.63 (0.27, 1.48) |  |
|  | Other | 0.40 (0.18, 0.89) |  |
| Prior relapse (vs None)^a^ | Yes | 2.46 (1.53, 3.96) | < 0.001 |
| Child’s age | (Non-parametric – see Supplementary Figure 2) | | 0.578 |
| Site^b^ | (Global test) | | 0.035 |

^a^Relapse prior to enrolling in PRISM.

^b^We were not interested in differences between particular sites; but rather, included the variable in the model to adjust for observed and unobserved differences.

*Note*. CI: Confidence Interval; OR: Odds Ratio; *p*: p value


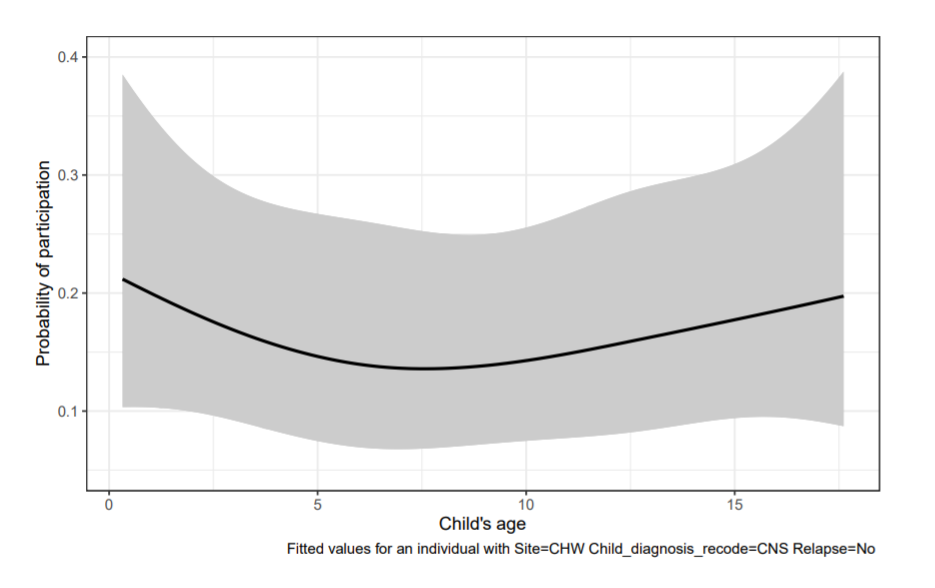
 **Supplementary Figure 2.** Estimated association between child’s age and probability of parental participation in PRISM-IMPACT

Considering possible differences between participating and non-participating parents, there was some evidence to suggest parents’ participation in PRISM-Impact differed by the child’s cancer type, with parents of children with a CNS diagnosis having the highest participation rate (*p*=0.049). After adjusting for the child’s age, hospital and cancer type, evidence suggested that parents whose child relapsed prior to enrolling in PRISM had a higher participation rate than parents whose child had not relapsed (*p*< 0.001; Supplementary Table 2, Figure 2).
