# Supplementary material for: Parents’ expectations, preferences, and recall of germline findings in a childhood cancer precision medicine trial
Source: Cancer. 2023 Jun 29;129(22):3620–32. doi: 10.1002/cncr.34917 (PMC10952780; doi:10.1002/cncr.34917)
Supplement: Supplementary file 2 — Figure S1 [file CNCR-129-3620-s003.docx]

**
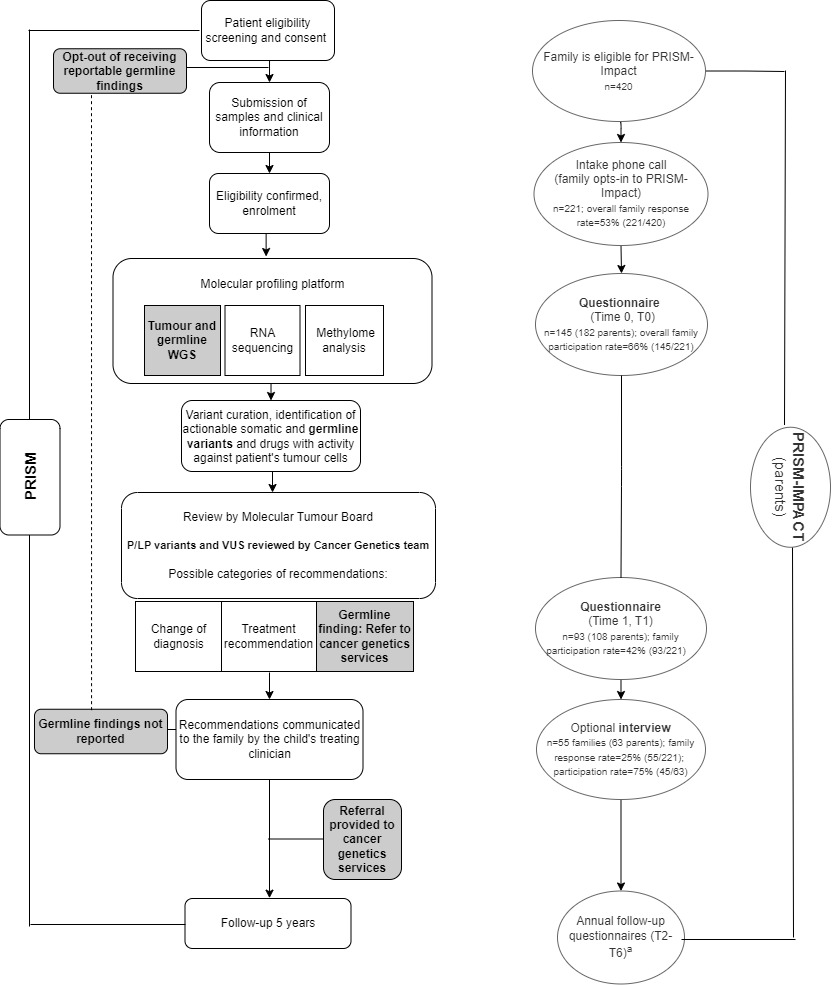
Supplementary Figure 1**. PRISM and PRISM-Impact study procedures, with procedures related to germline genome sequencing highlighted

^a^T2-T6 data not presented in this manuscript as data collection is ongoing.

*Note*. P/LP=pathogenic/likely pathogenic; VUS=variant of uncertain significance.
