# Supplementary material for: Parents’ expectations, preferences, and recall of germline findings in a childhood cancer precision medicine trial
Source: Cancer. 2023 Jun 29;129(22):3620–32. doi: 10.1002/cncr.34917 (PMC10952780; doi:10.1002/cncr.34917)
Supplement: Supplementary file 3 — Table S1 [file CNCR-129-3620-s001.docx]

**Supplementary Table 1.** Semi-structured interview guide

| Participant group | Questions |
| --- | --- |
| All parents | You may have received information from PRISM about your child’s genes that can ‘run in families’ or be passed from one generation to another. Can you tell me whether you received this kind of finding from PRISM? |
| Parents who reported that their child received a clinically-relevant germline finding from PRISM | I was hoping you could share what the experience of receiving the results was like for you, your child, and your family?  *Prompts:*  What do you understand about the results of the testing and the implications of it, if any? What about your child?  Has knowing these test results changed how you think about your child’s healthcare?  Has knowing these results changed how you think about your cancer risk?  How are you feeling about your child’s results now?  How have you and your family adjusted to this result?  Did you receive a referral to see a geneticist/genetic counsellor?  Did you end up seeing a geneticist/genetic counsellor about your child’s results?  Thinking about your experience, what do you think in general are the advantages/disadvantages of genetic counselling and/or testing in children with cancer?  Now that you have been through the process, was there anything your child’s medical team and/or cancer genetics services could have improved? |
